# A Novel EphA2 Inhibitor Exerts Beneficial Effects in PI-IBS *in Vivo* and *in Vitro* Models via Nrf2 and NF-κB Signaling Pathways

**DOI:** 10.3389/fphar.2018.00272

**Published:** 2018-03-27

**Authors:** Li Zeng, Kaixue Li, Hong Wei, Jingjing Hu, Lu Jiao, Shaoyong Yu, Ying Xiong

**Affiliations:** ^1^Department of Gastroenterology, The First Affiliated Hospital of Shenzhen University, The Second People’s Hospital of Shenzhen, Shenzhen, China; ^2^Division of Gastroenterology and Hepatology, Johns Hopkins University School of Medicine, Baltimore, MD, United States

**Keywords:** post-infectious irritable bowel syndrome, oxidative stress, inflammation, EphA2, nuclear factor-erythroid 2-related factor 2, NF-κB

## Abstract

Though the detailed pathological mechanism of post-infectious irritable bowel syndrome (PI-IBS) remains unclear, accumulating evidence indicates that oxidative stress and inflammation are implicated in the process of PI-IBS. Oxidative stress and inflammation are regulated by Nrf2 and NF-κB signaling pathways, respectively. EphA2, a member of Eph receptor family, promotes oxidative stress and inflammatory responses via regulation of Nrf2 and NF-κB signaling pathways in various types of human diseases. Understanding the mechanisms by which EphA2 regulate oxidative stress and inflammation in PI-IBS is important for the development of new strategies to treat PI-IBS. However, the effects of ALW-II-41-27, a novel EphA2 inhibitor on PI-IBS and the underlying molecular mechanisms have never been studied. In the present study, we showed that ALW-II-41-27 decreased gastrointestinal motility and abdominal withdrawal reflex (AWR) scores, markedly reduced the levels of oxidative stress markers [4-hydroxy-2-nonenal (4-HNE), protein carbonyl, and 8-hydroxy-2-de-axyguanine (8-OHdG)] and proinflammatory cytokines (TNF-α, IL-6, IL-17, and ICAM-1), and remarkably increased the level of anti-inflammatory cytokine (IL-10) in serum and colon of *Trichinella spiralis*-infected mice. Moreover, ALW-II-41-27 was effective in suppressing oxidative stress and inflammation in LPS-treated NCM460 colonic cells. Treatment of ALW-II-41-27 reversed the activation of NF-κB and inactivation of Nrf2 in LPS-treated NCM460 cells. Importantly, these protective effects of ALW-II-41-27 were partially inhibited by EphA2 KO and abolished by EphA2 overexpression. In conclusion, EphA2 may represent a promising therapeutic target for patients with PI-IBS and ALW-II-41-27 might function as a novel therapeutic agent for PI-IBS.

## Introduction

Irritable bowel syndrome (IBS) is one of the most prevalent functional gastrointestinal disorders (Oświȩcimska et al., 2017). It is characterized by the presence of recurrent or chronic abdominal pain or discomfort and bloating (Mearin et al., 2016). Three and thirty-five percentage patients with IBS develop PI-IBS after acute gastrointestinal infection (Sundin et al., 2015). Patients with PI-IBS have greater colonic hypercontractility than IBS (Kanazawa et al., 2014). Although it is not a life-threatening disease, PI-IBS jeopardizes the quality of life concerns and remains a substantial burden on the health care system (Kanuri et al., 2016).

Though the detailed pathological mechanism of PI-IBS remains unclear, accumulating evidence indicates that oxidative stress and inflammation are implicated in the process of PI-IBS (Long et al., 2016; Choghakhori et al., 2017). Oxidative stress is an imbalance between reactive oxygen species production and the endogenous antioxidant enzyme system (Cramer et al., 2017). Excessive reactive oxygen species causes lipid peroxidation and initiate damage to protein and DNA, leading to production of 4-HNE, protein carbonyl, and 8-OHdG (Kołodziej et al., 2017). In particular, an imbalance of pro-inflammatory cytokines (TNF-α, IL-6, IL-17, and ICAM-1) and anti-inflammatory cytokines (IL-10) may play a key role in the local intestinal inflammation (Charrad et al., 2016). The interaction of oxidative stress and low-grade inflammation together aggravate the symptoms of PI-IBS.

Oxidative stress and inflammation are regulated by Nrf2 and NF-κB signaling pathways, respectively. NF-κB signaling is well-known for its roles in regulation of inflammation (Yang et al., 2016). HO-1 is a critical phase II antioxidant enzyme that participates in suppression of oxidative stress (Rizzardini et al., 2016). Transcriptional activity and expression of HO-1 are regulated by Nrf2 signaling (Loboda et al., 2016). Previous studies investigated the association between Nrf2 and NF-κB signaling pathway. Activation of Nrf2 signaling pathway could inhibit NF-κB signaling pathway (Cuadrado et al., 2014; Velagapudi et al., 2017).

Understanding the mechanisms by which erythropoietin-producing hepatocellular (Eph) tyrosine kinase receptors regulate oxidative stress and inflammation is important for the development of new strategies to treat PI-IBS. The Eph receptor family is evolutionarily conserved and the largest of the receptor tyrosine kinase families (Park and Lee, 2015). Eph receptor family can be divided into A or B subgroups, based on their specific affinities for different subsets of ephrin ligands (Barquilla and Pasquale, 2015). Eph receptors play a central role in contact-dependent communication between cells, oxidative stress, inflammatory response and IBS (Peng and Chen, 2010; Singh et al., 2012; Takasugi et al., 2017; Kang et al., 2018). EphA2, a member of Eph receptor family, promotes oxidative stress and inflammatory responses in various types of human diseases (Chan and Sukhatme, 2009; Okazaki et al., 2009; Carpenter et al., 2012; Funk et al., 2012; Takasugi et al., 2017). Importantly, EphA2 was reported to be a modulator of several signaling pathways, including PI3K-Akt-NF-κB, Src-NF-κB, Nrf2, E-cadherin, and mTOR (Carpenter et al., 2012; Hong et al., 2016). ALW-II-41-27, a novel ATP-competitive EphA2 inhibitor (Amato et al., 2014), has been proved to inhibit type II RET tyrosine kinase and promote apoptosis in tumor cells (Moccia et al., 2015). Since oxidative stress and inflammation play a pivotal role in PI-IBS, we hypothesized ALW-II-41-27 might exert beneficial effects on PI-IBS.

In the present study, we found that EphA2 was elevated in *Trichinella spiralis*-infected mice and LPS-treated human intestinal epithelial cells. EphA2 inhibitors ALW-II-41-27 were effective in inhibiting oxidative stress and inflammation through activating Nrf2 and suppressing NF-κB signaling pathways.

## Materials and Methods

### Reagents

ALW-II-41-27 (Purify ≥ 99.3%) were purchased from MedChem Express (Monmouth Junction, NJ, United States). *Escherichia coli* LPS (*E. coli* 0127: B8), charcoal and gum acacia were purchased from Sigma-Aldrich (St. Louis, MO, United States). EphA2 CRISPR/Cas9 KO plasmid (sc-400535-KO-2), HDR plasmid (sc-400535), Control CRISPR/Cas9 plasmid (sc-418922), EphA2 CRISPR activation plasmid (sc-400535-ACT), Control CRISPR activation plasmid (sc-437275), UltraCruz^®^ transfection reagent (sc-395739), puromycin dihydrochloride (sc-108071), nuclear protein and tissue protein extraction kits were purchased from Santa Cruz Biotechnology (Santa Cruz, CA, United States). The bicinchoninic acid assay kits and the enhanced chemiluminescence Western blotting detection kits were purchased from Pierce Biotechnology (Rockford, IL, United States). Foetal bovine serum (FBS), Dulbecco’s Modified Eagle Medium (DMEM), penicillin and streptomycin were purchased from GIBCO BRL (Grand Island, NY, United States). ELISA kits were obtained from Thermo Fisher Scientific (Sunnyvale, CA, United States).

### Animals and Treatments

Male C57BL/6 mice (18–20 g) were purchased from Beijing Vital River Laboratory Animal Technology Co., Ltd. (Beijing, China). This study was carried out in accordance with the recommendations of the National Institutes of Health Guidelines on the Use and Care of Animals and the Institutional Animal Experiment Committee of the Second People’s Hospital of Shenzhen. The protocol was approved by the Institutional Animal Experiment Committee of the Second People’s Hospital of Shenzhen. All efforts have been made to minimize animal suffering and the number of animals used. Mice were housed in a sterile animal room of laboratory and maintained under the following conditions: the temperature of 23 ± 2°C, the humidity of 65 ± 5%, and a 12 h/12 h light/dark cycle. The animals were provided with food and water *ad libitum*. After 7 days of environmental adaption, 60 mice were randomly divided into six groups (*n* = 10 per group): control group, PI-IBS model group, and ALW-II-41-27 (12.5, 25, 50, and 100 μg/kg) treatment group. To induce *Trichinella spiralis* mouse model of PI-IBS, the mice were infected with *Trichinella spiralis* larvae (300 larvae per mouse) by oral gavage (0.2 ml in saline) (Kalia et al., 2008). The mice in ALW-II-41-27 treatment group were administered intraperitoneally with ALW-II-41-27 at doses of 12.5, 25, 50, or 100 μg/kg once daily for 7 consecutive days. Control and PI-IBS model groups were administered with saline. Before 1 day and after 1 day of medication, body weight and fecal water content of all mice were measured.

### Gastrointestinal Motility

Gastrointestinal motility was assessed by feeding mice charcoal meals and determining the distance traveled by the charcoal in a fixed amount of time (Li et al., 2006). Prior to measuring gastrointestinal motility, mice (*n* = 10 per group) were fasted overnight and given 0.2 ml 10% charcoal in 5% gum acacia by oral gavage. Mice were euthanized 30 min later and the intestinal tracts were carefully removed. The total length from the pylorus to the cecum was measured. Gastrointestinal motility is expressed as the percentage of the total intestinal length traversed by the charcoal.

### AWR Scores

Visceral sensitivity of colorectal distention in mice was assessed by AWR scores at day 14 infection (Yang et al., 2015). Briefly, mice (*n* = 10 per group) were anesthetized with ether, a latex double-lumen catheter attached to a balloon dilator (6-Fr, 2 mm external diameter) was used. The balloon was vaseline-coated and inserted rectally into the descending colon. After waking up and adapting for 1 h, colorectal distention was performed. Each 20 s distention was followed by a 30 s resting period. Distention was repeated three times, and the balloon was deflated and withdrawn after assessing AWR. AWR responses were measured by blind observers: 0, no behavioral response to colorectal distention; 1, simple head movement followed by immobility; 2, contraction of abdominal muscles; 3, lifting of the abdomen; and 4, arching of body and lifting of pelvic structures. Colorectal distention was calculated as the amount of injected water when the AWR score was 3.

### Cell Culture and Drug Treatment

NCM460 colonic cells were obtained from the Cell Resource Center of the Institute of Basic Medical Sciences, Peking Union Medical College and Chinese Academy of Medical Sciences (Beijing, China) NCM460 cells were cultured in DMEM supplemented with FBS and 1% penicillin (100 U/mL)/streptomycin (100 mg/mL) at 37°C in 5% CO_2_ and 95% atmosphere (Lu et al., 2016). To induce the cellular model of PI-IBS. NCM460 cells were incubated with ALW-II-41-27 (50, 100, 200, and 400 ng/mL) for 24 h, and then treated with LPS (50 ng/mL) for 24 h. Cell viability was evaluated with the Cell Counting Kit-8 (CCK-8). The release of lactate dehydrogenase (LDH) was detected using the assay kit (Nanjing Jiancheng Bioengineering Institute, China).

### Stable Transfection

To establish EphA2 gene knockout cells, NCM460 cells (1 × 10^5^ cells/well) were seeded in six-well plates, when 60% confluence was reached, cells were co-transfected with EphA2 CRISPR/Cas9 KO plasmid or Control CRISPR/Cas9 plasmid and EphA2 HDR Plasmid in antibiotic-free growth medium with UltraCruz^®^ Transfection Reagent (Santa Cruz, CA, United States) and incubate overnight. EphA2 CRISPR/Cas9 KO Plasmid is designed to disrupt gene expression by causing a double-strand break (DSB) in a 5′ constitutive exon within the EphA2 (human) gene. EphA2 CRISPR/Cas9 KO Plasmid (h2) consists of a pool of 3 plasmids, each encoding the Cas9 nuclease and a target-specific 20 nt guide RNA (gRNA) designed for maximum knockout efficiency. Stable EPHA2 KO cell clones were selected with media containing puromycin dihydrochloride (5 μg/mL, Sigma-Aldrich). Western blotting was used for monitoring of EPHA2 (human) gene expression prior to and after knockout.

To establish EphA2 gene overexpression cells, NCM460 cells (1 × 10^5^ cells/well) were seeded in six-well plates, when 60% confluence was reached, cells were transfected with EphA2 CRISPR activation plasmid or Control CRISPR activation plasmid in antibiotic-free growth medium with UltraCruz^®^ Transfection Reagent (Santa Cruz, CA, United States) and incubate overnight. EphA2 CRISPR Activation Plasmid consist of the following 3 plasmids at a 1:1:1 mass ratio: the CRISPR/dCas9-VP64-Blast plasmid encoding the deactivated Cas9 (dCas9) nuclease (D10A and N863A) fused to the transactivation domain VP64, and a blasticidin resistance gene; the MS2-P65-HSF1-Hygro plasmid encoding the MS2-p65-HSF1 fusion protein, and a hygromycin resistance gene; the sgRNA (MS2)-Puro plasmid encoding a target-specific 20 nt gRNA, and a puromycin resistance gene. The sgRNA (MS2)-Puro plasmids in EphA2 CRISPR Activation Plasmid (h) and EphA2 CRISPR Activation Plasmid (h2) each encode their own, unique, target-specific 20 nt gRNA. The resulting SAM complex provides a robust transcription activation system for the upregulation of EphA2. Western blotting was used for monitoring of EPHA2 gene expression prior to and after activation. NCM460 cells were incubated with ALW-II-41-27 (100 ng/mL) for 24 h, and then treated with LPS (50 ng/ml) for 24 h.

### ELISA

The mice (*n* = 10 per group) were humanely sacrificed (ether inhalation and cervical dislocation) on day 14 post-infection. Serum was collected and stored at -80°C. Proximal colons (4 cm in length; 1–2 cm away from caecum) were collected and flushed with saline to remove gut contents and immediately preserved in liquid nitrogen. The colons were homogenized in RIPA buffer containing phosphatase inhibitor and complete protease inhibitor cocktail (Takara, Japan). The homogenates were then centrifuged at 10000 rpm for 20 min, and protein concentrations in the supernatant of homogenates were determined using a bicinchoninic acid assay kit. NCM460 cells washed, trypsinized, and lysed in RIPA buffer containing phosphatase inhibitor and complete protease inhibitor cocktail. The homogenates were then centrifuged at 10000 rpm for 20 min, and protein concentrations in the supernatant of homogenates were determined using a bicinchoninic acid assay kit. The levels of 4-HNE, protein carbonyl, 8-OHdG, and HO-1 activity as well as TNF-α, IL-6, IL-10, IL-17, ICAM-1, and NF-κB activity in cells, serum and the colons were measured by technicians who were blinded to the experimental groups using ELISA kits. At least three independent experiments were carried out.

### Western Blot Analysis

Western blot analysis was performed by technicians who were blinded to the experimental groups. Briefly, the protein extracts of colon (*n* = 10 per group) were loaded into 8–12% Bis-Tris gels in a Bio-Rad slab gel apparatus (Bio-Rad, Hercules, CA, United States) and electrophoretically transferred to a nitrocellulose membrane. Blots were probed with the following antibodies: EphA2 (ab5386), Nrf2 (ab62352), NF-κB p65 (ab16502), β-actin (ab8227), and Lamin B (ab194109) were obtained from Abcam and were used at a 1:1,000 dilution, followed by the appropriate secondary antibodies. Bands were visualized using the enhanced chemiluminescence Western blotting detection kits, scanned with a densitometer (Bio-Rad) and analyzed quantitatively with commercial equipment (Multi-Analyst Macintosh Software for Image Analysis Systems; Bio-Rad).

### Statistical Analysis

All experiments were performed a minimum of three times. Data are presented as Mean ± SD. One-way ANOVA was used to assess significant differences for multiple groups, followed by *post hoc* Bonferroni’s test. *P* < 0.05 was considered statistically significant.

## Results

### ALW-II-41-27 Prevented Intestinal Dysmotility in *Trichinella spiralis*-Infected Mice

**FIGURE 1:**
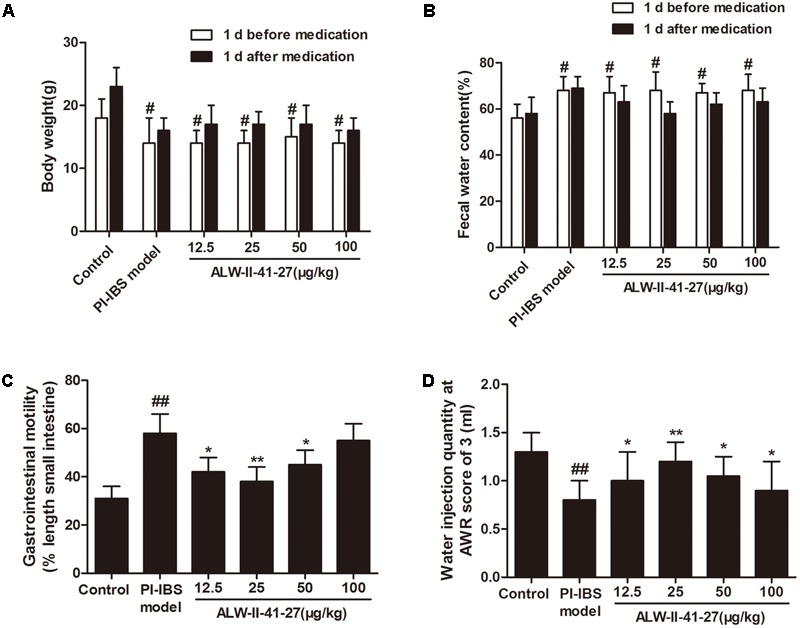
ALW-II-41-27 intestinal dysmotility in *Trichinella spiralis*-infected mice. Effects of ALW-II-41-27 on body weight **(A)**, fecal water content **(B)**, gastrointestinal motility **(C)** and AWR scores **(D)** in *Trichinella spiralis*-infected mice. The results are expressed as the mean ± SD (*n* = 10 per group); ^#^indicates a significant difference from control group (*P* < 0.05); ^##^indicates a significant difference from control group (*P* < 0.01); ^∗^indicates a significant difference from PI-IBS group (*P* < 0.05); ^∗∗^indicates a significant difference from PI-IBS group (*P* < 0.01).

As shown in **Figures 1A,B**, ALW-II-41-27 (12.5, 25, 50, and 100 μg/kg) treatment for 7 days did not affect the body weight and fecal water content of *Trichinella spiralis*-infected mice (*P* > 0.05). At day 14 infection, gastrointestinal motility was assessed by feeding mice charcoal meals and determining the distance traveled by the charcoal in a fixed amount of time and visceral sensitivity of colorectal distention in mice was assessed by AWR scores. Gastrointestinal motility was significantly increased in *Trichinella spiralis*-infected mice compared with control group (*P* < 0.01, **Figure 1C**). AWR scores in the PI-IBS group were significantly higher than those in the control group (*P* < 0.01, **Figure 1D**). Compared with PI-IBS model group, treatment of ALW-II-41-27 (12.5, 25, and 50 μg/kg) for 7 days significantly decreased gastrointestinal motility and AWR scores (*P* < 0.01). Moreover, ALW-II-41-27 showed the best protection at 25 μg/kg body weight.

### ALW-II-41-27 Inhibited Oxidative Stress and Inflammation *in Vivo* and *in Vitro*

**FIGURE 2:**
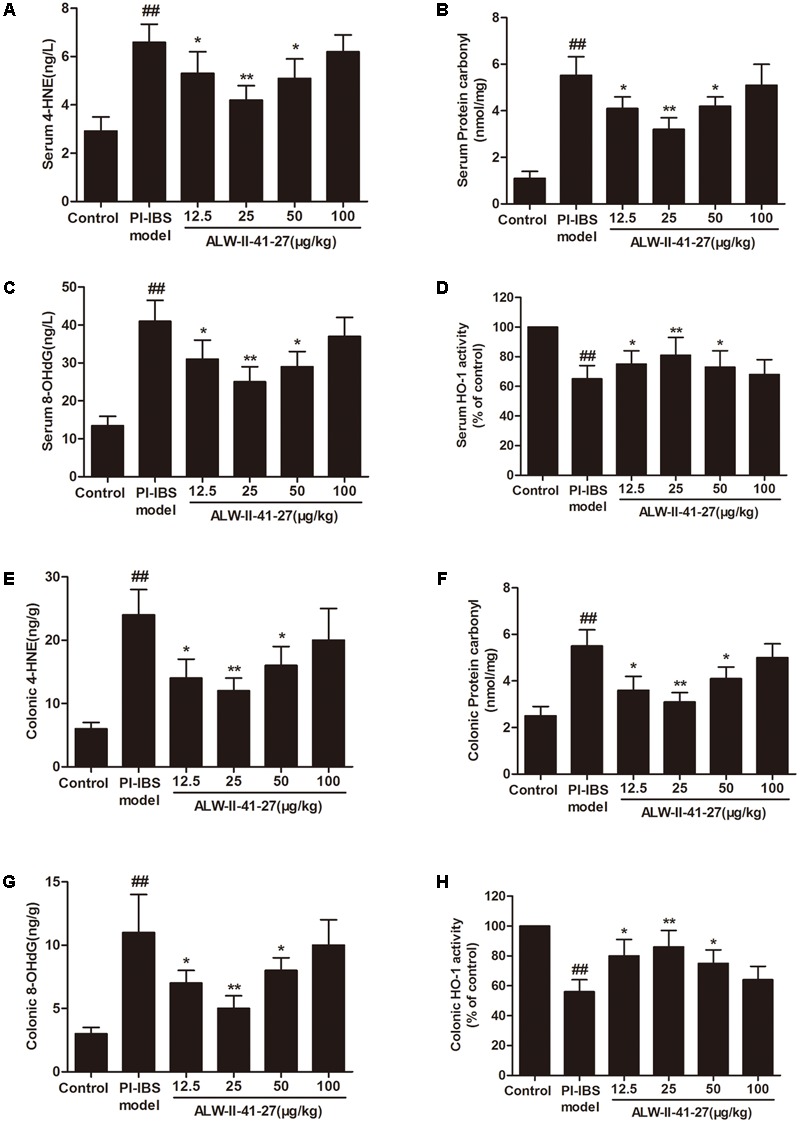
ALW-II-41-27 suppressed oxidative stress in *Trichinella spiralis*-infected mice. Effects of ALW-II-41-27 on the levels of serum 4-HNE **(A)**, serum protein carbonyl **(B)**, serum 8-OHdG **(C)**, serum HO-1 activity **(D)**, colonic 4-HNE **(E)**, colonic protein carbonyl **(F)**, colonic 8-OHdG **(G)**, colonic HO-1 activity **(H)**. The results are expressed as the mean ± SD (*n* = 10 per group); ^##^indicates a significant difference from control group (*P* < 0.01); ^∗^indicates a significant difference from PI-IBS group (*P* < 0.05); ^∗∗^indicates a significant difference from PI-IBS group (*P* < 0.01).

**FIGURE 3:**
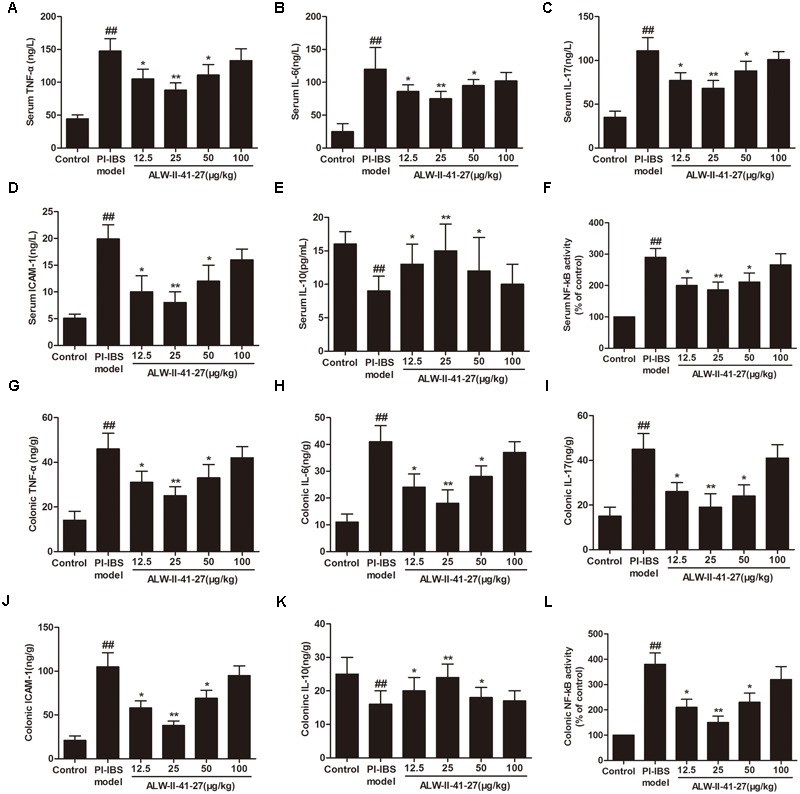
ALW-II-41-27 inhibited inflammation in *Trichinella spiralis*-infected mice. Effects of ALW-II-41-27 on the expression of serum TNF-α **(A)**, serum IL-6 **(B)**, serum IL-17 **(C)**, serum ICAM-1 **(D)**, serum IL-10 **(E)** and serum NF-κB activity **(F)**, colonic TNF-α **(G)**, colonic IL-6 **(H)**, colonic IL-17 **(I)**, colonic ICAM-1 **(J)**, colonic IL-10 **(K)** and colonic NF-κB activity **(L)**. The results are expressed as the mean ± SD (*n* = 10 per group); ^##^indicates a significant difference from control group (*P* < 0.01); ^∗^indicates a significant difference from PI-IBS group (*P* < 0.05); ^∗∗^indicates a significant difference from PI-IBS group (*P* < 0.01).

**FIGURE 4:**
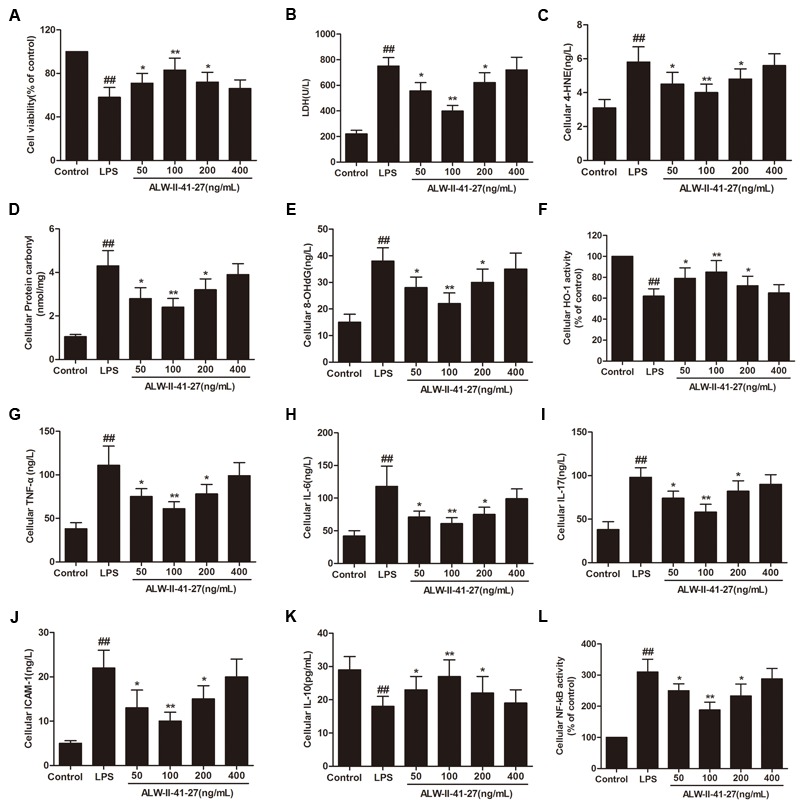
ALW-II-41-27 inhibited LPS-induced oxidative stress and inflammation in NCM460 cells. Effects of ALW-II-41-27 on cell viability **(A)**, LDH leakage **(B)**, cellular 4-HNE **(C)**, cellular protein carbonyl **(D)**, cellular 8-OHdG **(E)**, cellular HO-1 activity **(F)**, cellular TNF-α **(G)**, cellular IL-6 **(H)**, cellular IL-17 **(I)**, cellular ICAM-1 **(J)**, cellular IL-10 **(K)** and cellular NF-κB activity **(L)**. ^##^Indicates a significant difference from control cells (*P* < 0.01); ^∗^indicates a significant difference from LPS group (*P* < 0.05); ^∗∗^indicates a significant difference from LPS group (*P* < 0.01).

Oxidative stress and inflammation play pivotal roles in the occurrence and persistence of PI-IBS symptoms (Yang et al., 2016). In the current study, the levels of oxidative stress and inflammation markers were detected by ELISA in serum and colon tissue lysates of *Trichinella spiralis*-infected mice as well as in NCM460 cells treated with LPS in the presence or absence of ALW-II-41-27. As shown in **Figures 2A–H**, compared with control group, *Trichinella spiralis* infection significantly increased the levels of 4-HNE, protein carbonyl, 8-OHdG, and significantly decreased HO-1 activity. However, treatment of ALW-II-41-27 (12.5, 25, and 50 μg/kg) markedly increased the activity of HO-1 and significantly decreased the levels of 4-HNE, protein carbonyl, and 8-OHdG in *Trichinella spiralis*-infected mice. Moreover, ALW-II-41-27 showed the best protection at 25 μg/kg body weight (Traini et al., 2016). As shown in **Figures 3A–L**, *Trichinella spiralis* infection markedly enhanced the levels of proinflammatory cytokines (TNF-α, IL-6, IL-17, and ICAM-1, *P* < 0.01) but remarkably reduced the level of anti-inflammatory cytokine (IL-10) in colon, compared with control group (*P* < 0.01). Treatment of ALW-II-41-27 (12.5, 25, and 50 μg/kg) significantly reduced TNF-α,IL-6, IL-17, and ICAM-1, and significantly increased the level of IL-10, compared with PI-IBS model group (*P* < 0.01). Moreover, *Trichinella spiralis* infection significantly increased NF-κB activity (*P* < 0.01). However, treatment of ALW-II-41-27 (12.5, 25, and 50 μg/kg) inhibited the enhanced activity of NF-κB in *Trichinella spiralis*-infected mice (*P* < 0.01) and ALW-II-41-27 showed the best protection at 25 μg/kg body weight. Moreover, LPS treatment significantly decreased the cell viability and increased LDH leakage in NCM460 cells (**Figures 4A,B**). The levels of oxidative stress markers (4-HNE, protein carbonyl, 8-OHdG) and inflammation markers (TNF-α, IL-6, IL-17, and ICAM-1) as well as the activity of NF-κB were significantly increased after LPS treatment, compared with the control (*P* < 0.05,). Meanwhile, decreased HO-1 activity and reduced IL-10 level were observed in LPS-treated NCM460 cells, compared with the control (*P* < 0.05, **Figures 4C–L**). However, ALW-II-41-27 significantly reversed LPS-induced oxidative stress and inflammation in NCM460 cells (*P* < 0.05).

### ALW-II-41-27 Activated Nrf2 and Inhibited NF-κB Signaling *in Vivo* and *in Vitro*

**FIGURE 5:**
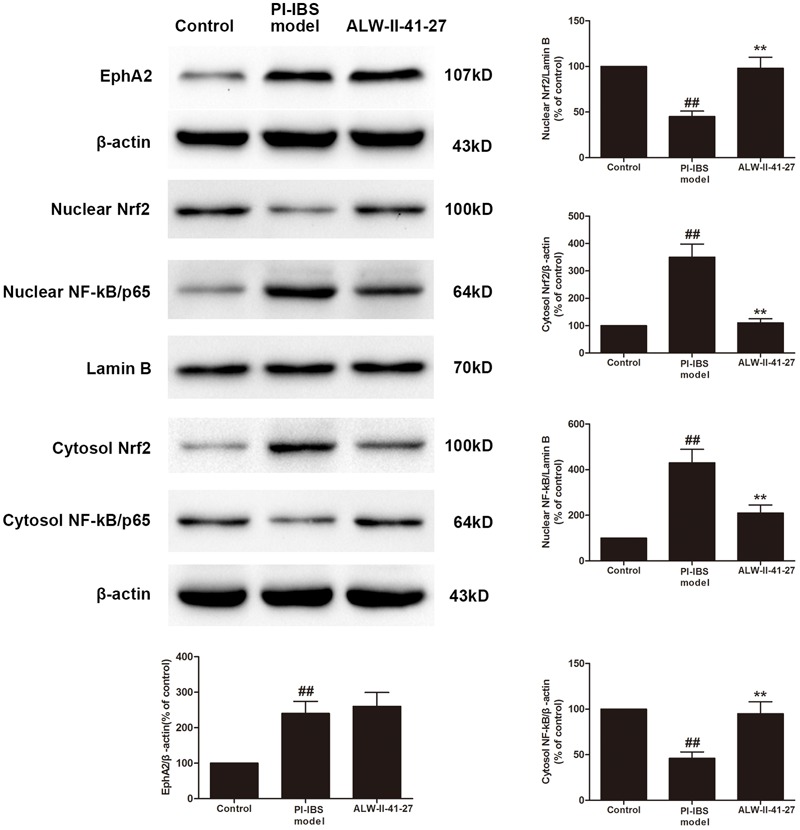
ALW-II-41-27 activated Nrf2 signaling whereas inhibited NF-κB signaling in *Trichinella spiralis*-infected mice. The protein expressions of EphA2, nuclear Nrf2, cytosol Nrf2, and nuclear NF-κB/p65, cytosol NF-κB/p65, Lamin B, and β-actin in colon tissue lysates were measured by western blotting and densitometry analysis. The results are expressed as the mean ± SD (*n* = 10 per group); ^##^indicates a significant difference from control group (*P* < 0.01); ^∗∗^indicates a significant difference from PI-IBS group (*P* < 0.01).

**FIGURE 6:**
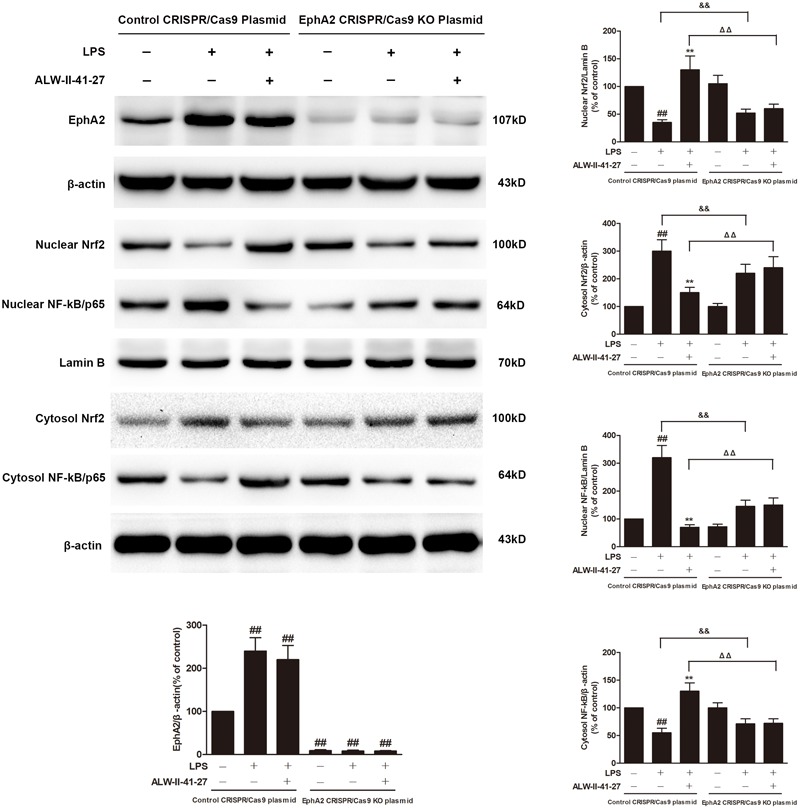
EphA2 knock-out partially inhibited the effects of ALW-II-41-27 on LPS-treated cells. NCM460 cells were transfected to obtain clones knock-out for EphA2. Control cells and EphA2 KO cells were treated for 24 h with ALW-II-41-27 (100 ng/mL) and then treated with LPS (50 ng/mL) for 24 h. The protein expressions of EphA2, nuclear Nrf2, cytosol Nrf2, and nuclear NF-κB/p65, cytosol NF-κB/p65, Lamin B and β-actin in colon tissue lysates were measured by western blotting and densitometry analysis. The results are expressed as the mean ± SD (*n* = 10 per group); ^##^indicates a significant difference from control cells (*P* < 0.01); ^∗∗^indicates a significant difference from LPS group (*P* < 0.01); ^ΔΔ^indicates a significant difference from control cells treated LPS; ^&&^indicates a significant difference from control cells treated with ALW-II-41-27 and LPS.

**FIGURE 7:**
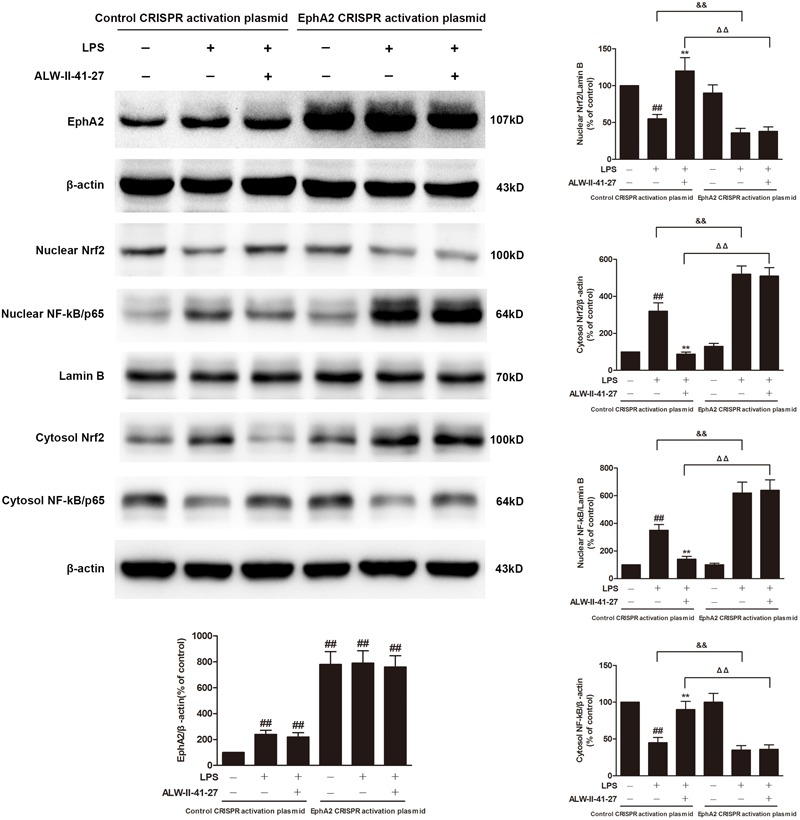
EphA2 overexpression abolished the effects of ALW-II-41-27 on LPS-treated cells. NCM460 cells were transfected to obtain clones overexpressing for EphA2. Control cells and EphA2 overexpressing cells were treated for 24 h with ALW-II-41-27 and then treated with LPS (50 ng/mL) for 24 h. The protein expressions of EphA2, nuclear Nrf2, cytosol Nrf2, and nuclear NF-κB/p65, cytosol NF-κB/p65, Lamin B and β-actin in colon tissue lysates were measured by western blotting and densitometry analysis. The results are expressed as the mean ± SD (*n* = 10 per group); ^##^indicates a significant difference from control cells (*P* < 0.01); ^∗∗^indicates a significant difference from LPS group (*P* < 0.01); ^ΔΔ^indicates a significant difference from control cells treated LPS; ^&&^indicates a significant difference from control cells treated with ALW-II-41-27 and LPS.

**FIGURE 8:**
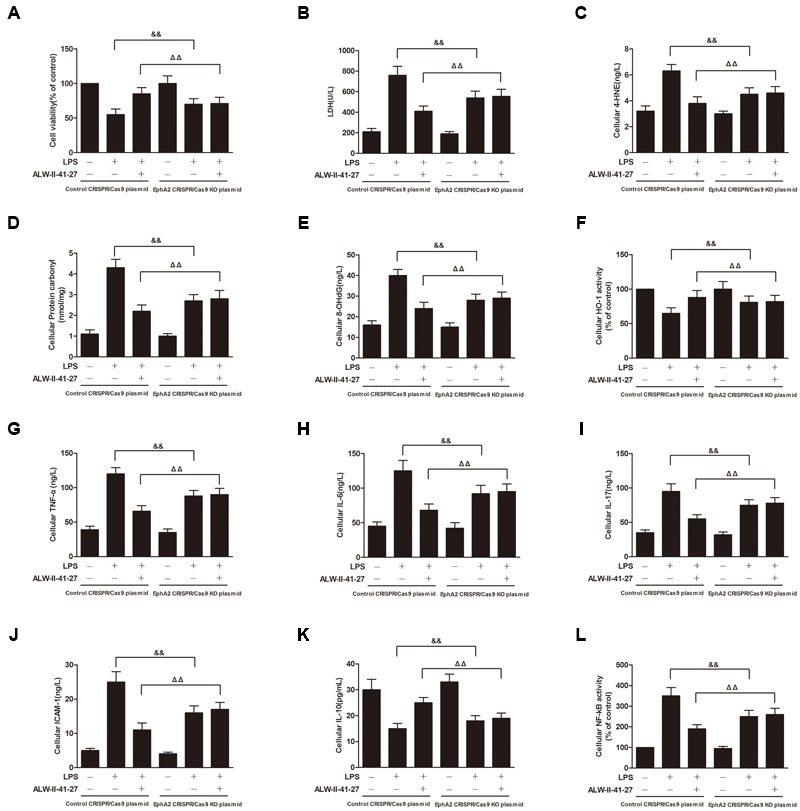
EphA2 knock-out partially inhibited the protective effects of ALW-II-41-27 on LPS-induced oxidative stress and inflammation. Effects of EphA2 knock-out on cell viability **(A)**, LDH leakage **(B)**, cellular 4-HNE **(C)**, cellular protein carbonyl **(D)**, cellular 8-OHdG **(E)**, cellular HO-1 activity **(F)**, cellular TNF-α **(G)**, cellular IL-6 **(H)**, cellular IL-17 **(I)**, cellular ICAM-1 **(J)**, cellular IL-10 **(K)** and cellular NF-κB activity **(L)**. ^##^Indicates a significant difference from control cells (*P* < 0.01); ^∗^indicates a significant difference from LPS group (*P* < 0.05); ^∗∗^indicates a significant difference from LPS group (*P* < 0.01); ^ΔΔ^indicates a significant difference from control cells treated LPS; ^&&^indicates a significant difference from control cells treated with ALW-II-41-27 and LPS.

**FIGURE 9:**
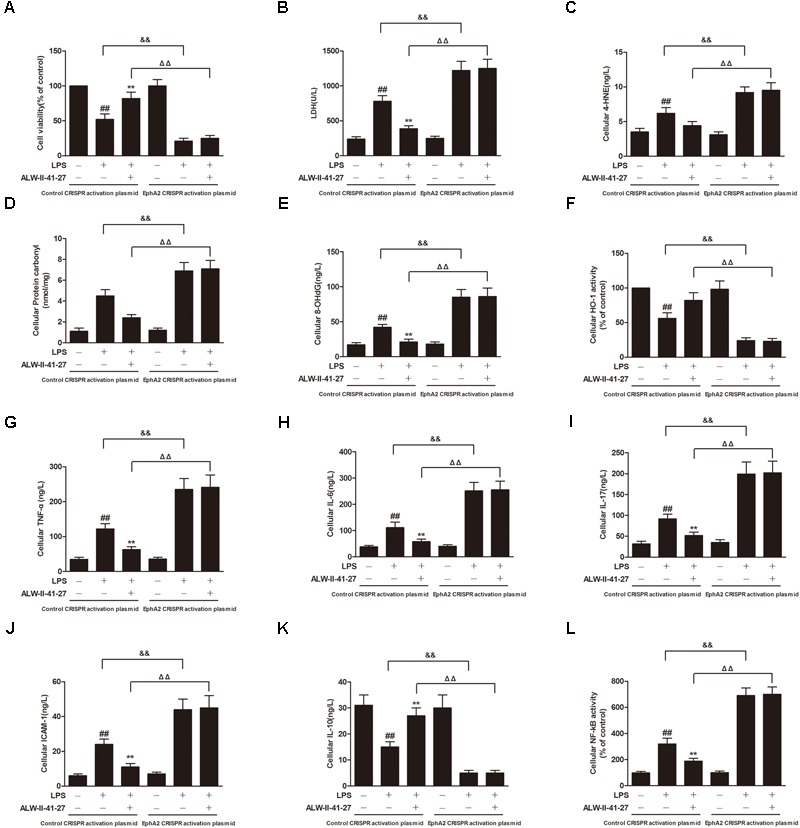
EphA2 overexpression abolished the protective effects of ALW-II-41-27 on LPS-induced oxidative stress and inflammation. Effects of EphA2 overexpression on cell viability **(A)**, LDH leakage **(B)**, cellular 4-HNE **(C)**, cellular protein carbonyl **(D)**, cellular 8-OHdG **(E)**, cellular HO-1 activity **(F)**, cellular TNF-α **(G)**, cellular IL-6 **(H)**, cellular IL-17 **(I)**, cellular ICAM-1 **(J)**, cellular IL-10 **(K)** and cellular NF-κB activity **(L)**. ^##^Indicates a significant difference from control cells (*P* < 0.01); ^∗^indicates a significant difference from LPS group (*P* < 0.05); ^∗∗^indicates a significant difference from LPS group (*P* < 0.01); ^ΔΔ^indicates a significant difference from control cells treated LPS; ^&&^indicates a significant difference from control cells treated with ALW-II-41-27 and LPS.

Eph A2 has been implicated in regulation of NF-κB signaling pathway (Carpenter et al., 2012; Hong et al., 2016). As shown in **Figure 5**, compared with control group, the protein expression of EphA2 was markedly increased in colon of *Trichinella spiralis*-infected mice. The nuclear NF-κB/p65 abundance was significantly increased but nuclear translocation of Nrf2 was significantly decreased in colon of *Trichinella spiralis*-infected mice. Compared with PI-IBS group, treatment of ALW-II-41-27 (25 μg/kg) significantly increased the level of nuclear Nrf2 and remarkably reduced the nuclear translocation of NF-κB in colon of ALW-II-41-27-treated mice (*P* < 0.01). However, the protein expression of EphA2 was not affected by treatment of ALW-II-41-27. Additionally, NCM460 cells were treated for 24 h with ALW-II-41-27 (100 ng/mL) and then treated with LPS (50 ng/mL) for 24 h. NCM460 cells were transfected to obtain clones KO or overexpressing for EphA2. The protein expression of EphA2, nuclear NF-κB/p65 and Nrf2 were shown in **Figures 6** and **7**, respectively. The protective effects of ALW-II-41-27 against LPS were shown in **Figures 6–9**. However, treatment of NCM460 cells with ALW-II-41-27 significantly reversed the activation of NF-κB and inhibition of Nrf2.

### EphA2 KO Partially Inhibited the Protective Effects of ALW-II-41-27

NCM460 cells were transfected to obtain clones knock-out for EphA2. As shown in **Figure 7**, knock-out of EphA2 was confirmed by western blot. Compared to control cells, EphA2 KO cells treated with LPS had a significant decrease in nuclear NF-κB/p65 and a significant increase in nuclear Nrf2. Subsequently, the levels of oxidative stress and inflammation markers were partially inhibited, meanwhile, the cell viability and LDH leakages were decreased (**Figure 8**). Interestingly, the protective effects of ALW-II-41-27 against LPS were also partially inhibited in EphA2 KO cells (**Figures 6, 8**).

### EphA2 Overexpression Abolished the Protective Effects of ALW-II-41-27

NCM460 cells were transfected to obtain clones overexpressing for EphA2. As shown in **Figure 8**, overexpression of EphA2 was confirmed by western blot. Compared to control cells, EphA2 overexpression enhanced the nuclear translocation of NF-κB/p65 and the decrease in nuclear Nrf2 which were induced by LPS. Subsequently, the levels of oxidative stress and inflammation biomarkers were elevated, meanwhile, the cell viability and LDH leakage were increased (**Figure 9**). Interestingly, the protective effects of ALW-II-41-27 against LPS were abolished in EphA2 overexpressing cells.

## Discussion

In the current study, we identified ALW-II-41-27, a novel ATP competitive EphA2 inhibitor, was capable of inhibiting intestinal motility in the mouse model mimicking diarrhea-predominant PI-IBS Symptoms. Treatment of ALW-II-41-27 (12.5, 25, and 50 μg/kg) for 7 days remarkably decreased gastrointestinal motility and visceral sensitivity in the *Trichinella spiralis*-infected mice. To the best of our knowledge, this was the first confirmation that EphA2 inhibitor could attenuate disorder of gastrointestinal function of PI-IBS.

Although the pathogenesis of PI-IBS is not well understood, a large body of evidence suggests that oxidative stress and inflammation play a pivotal role in the occurrence and persistence of its symptoms (Akiho et al., 2010; Kaji et al., 2016). The imbalance of reactive oxygen species and antioxidant enzymes as well as the imbalance of proinflammatory cytokines and anti-inflammatory cytokines play a key role in the development of PI-IBS (Szpetnar et al., 2012; Sundin et al., 2015). Consistent with these findings, we found that the levels of biomarkers of oxidative stress (4-HNE, protein carbonyl, and 8-OHdG), the expression of proinflammatory cytokines (TNF-α, IL-6, IL-17, and ICAM-1), and the activity of NF-κB were remarkably increased in the serum and colon of *Trichinella spiralis*-infected mice as well as LPS-treated NCM460 colonic cells. However, the activity of Phase II antioxidant enzyme HO-1 and the level of anti-inflammatory cytokine IL-10 were significantly declined in the serum and colon of *Trichinella spiralis*-infected mice as well as LPS-treated NCM460 colonic cells. Treatment of ALW-II-41-27 effectively suppressed oxidative stress and inflammation *in vivo* and *in vitro*. These results suggested that ALW-II-41-27 attenuated PI-IBS via inhibiting oxidative stress and inflammatory responses.

Interestingly, ALW-II-41-27 treatment had the protective effects in non-linear dose-dependent manner, and ALW-II-41-27 showed the best protective effect at 25 μg/kg body weight and 100 ng/mL. Eph receptors and ephrins are known to emanate bidirectional signals: forward signals that propagate in Eph-expressing cells through Eph kinase activity, and reverse signal that propagate in ephrin-expressing cells (Ivanov and Romanovsky, 2006). In this study, the expression of EphA2 was significantly increased in colon of *Trichinella spiralis*-infected mice and LPS-treated NCM460 colonic cells. High doses of ALW-II-41-27 might result in excessive EphA2 inhibition, which generated unexpected effects. These results indicated moderate inhibition of EphA2 might be beneficial for the treatment of PI-IBS, however, the precise mechanisms remain to be investigated.

EphA2 is regarded as a regulator of several signaling pathways, including PI3K-Akt-NF-κB, Src-NF-κB, Nrf2, E-cadherin, and mTOR (Carpenter et al., 2012; Kumar and Chandran, 2016). Downstream signaling pathways activated by EphA2 are associated with oxidative stress and inflammation (Coulthard et al., 2012; Ieguchi, 2015). It is widely confirmed that Nrf2 and NF-κB signaling pathways play a key role in oxidative stress and inflammatory response (Lampiasi and Montana, 2017). Therefore, we have focused these two signaling cascades to investigate the effects of EphA2 inhibition on *Trichinella spiralis*- or LPS-induced oxidative stress and inflammation. Activation of Nrf2 signaling is required for the efficient localization of Nrf2 to the promoter regions of antioxidant responsive element in nucleus (Loboda et al., 2016). Inhibition of NF-κB signaling is also required for the efficient blockade of NF-κB/p65 nuclear translocation (Carpenter et al., 2012). In this study, ALW-II-41-27 could facilitate the translocation of Nrf2 to nucleus and inhibit nuclear translocation of NF-κB/p65 in colon of *Trichinella spiralis*-infected mice and LPS-treated NCM460 colonic cells. These results demonstrated that ALW-II-41-27 suppressed oxidative stress and inflammation via activation of Nrf2 signaling pathway and inhibition of NF-κB signaling pathway.

Whether EphA2 is responsible for activation of NF-κB signaling pathway and inhibition of Nrf2 signaling pathway in PI-IBS remains to be investigated. In the present study, NCM460 cells were transfected to obtain clones KO or overexpressing for EphA2. We found that knockout of EphA2 in NCM460 colonic cells partially inhibited the activation of NF-κB and the inactivation of Nrf2 caused by LPS. Moreover, EphA2 overexpression enhanced the nuclear translocation of NF-κB/p65 and the decrease in nuclear Nrf2 induced by LPS. Subsequently, LPS-induced oxidative stress and inflammation were partially inhibited in EphA2 KO cells but enhanced in EphA2 overexpressing cells. Importantly, the protective effects of ALW-II-41-27 were partially inhibited by EphA2 KO and abolished by EphA2 overexpression. These results demonstrated that EphA2 regulates Nrf2 and NF-κB signaling pathways. Although previous studies demonstrated that activation of Nrf2 signaling pathway could inhibit NF-κB signaling pathway (Lu et al., 2016; Velagapudi et al., 2017), whether ALW-II-41-27 activated Nrf2 signaling pathway, which in turn inhibited NF-κB signaling pathway, and the underlying mechanism by which EphA2 inhibitor activated Nrf2 signaling pathway and inhibited of NF-κB signaling pathway remain to be investigated.

## Conclusion

Collectively, our results represent the first evidence that EphA2 receptor inhibitor ALW-II-41-27 exerted beneficial effects on PI-IBS *in vivo* and *in vitro* models. These protective effects were associated with the activation of Nrf2 signaling pathway and the inactivation of NF-κB signaling pathway. If these effects of ALW-II-41-27 are validated in clinical trials, it might be a promising agent for the prevention and treatment of PI-IBS.

## Author Contributions

LZ, KL, HW, and YX contributed to the conception of the study. LZ, KL, JH, and YX contributed significantly to analysis and manuscript preparation. LZ, KL, LJ, and YX performed the data analyses and wrote the manuscript. LZ, KL, SY, and YX helped to perform the analysis with constructive discussions.

## Conflict of Interest Statement

The authors declare that the research was conducted in the absence of any commercial or financial relationships that could be construed as a potential conflict of interest.

## Abbreviations

4-HNE: 4-hydroxy-2-nonenal
8-OHdG: 8-hydroxy-2-de-axyguanine
AWR: abdominal withdrawal reflex
HO-1: heme oxygenase-1
Nrf2: nuclear factor-erythroid 2-related factor 2
PI-IBS: post-infectious irritable bowel syndrome
